# Digitally engaging participants in cancer clinical trials: Design and pilot of the Alliance Participant Engagement Portal (PEP)

**DOI:** 10.1093/jncics/pkag038

**Published:** 2026-04-11

**Authors:** Norah L Crossnohere, Nancy Campbell, Electra D Paskett, Marie E Wood, Rachel A Freedman, Anna Revette, Brett Nava-Coulter, Alvaro Alencar, Patricia A Spears, Vernal Branch, Andre Quina, Nareesa Mohammed-Rajput, Christine Duong, Steven Piantadosi, Suzanne George

**Affiliations:** Division of General Internal Medicine, Department of Internal Medicine, College of Medicine, The Ohio State University, Columbus, OH, United States; Alliance for Clinical Trials in Oncology Data Innovation Lab, Boston, MA, United States; Division of Cancer Prevention and Control, Department of Internal Medicine, College of Medicine, The Ohio State University, Columbus, OH, United States; Division of Epidemiology, College of Public Health, The Ohio State University, Columbus, OH, United States; University of Colorado Cancer Center, Aurora, CO, United States; Alliance for Clinical Trials in Oncology Data Innovation Lab, Boston, MA, United States; Dana-Farber Cancer Institute, Boston, MA, United States; Dana-Farber Cancer Institute, Boston, MA, United States; Dana-Farber Cancer Institute, Boston, MA, United States; Division of Hematology, Department of Medicine, University of Miami Sylvester Comprehensive Cancer Center, Miami, FL, United States; University of North Carolina at Chapel Hill, Chapel Hill, NC, United States; UNC Lineberger Comprehensive Cancer Center, Chapel Hill, NC, United States; The MITRE Corporation, Bedford, MA, United States; The MITRE Corporation, Bedford, MA, United States; The MITRE Corporation, Bedford, MA, United States; Brigham and Women’s Hospital, Boston, MA, United States; Alliance for Clinical Trials in Oncology Data Innovation Lab, Boston, MA, United States; Department of Medical Oncology, Dana-Farber Cancer Institute, Boston, MA, United States; Harvard Medical School, Boston, MA, United States

## Abstract

**Background:**

Digital tools may enhance clinical trial engagement by improving information flow and supporting participant-directed communication. The Alliance for Clinical Trials in Oncology developed the Participant Engagement Portal (PEP), a web-based platform for bidirectional communication and participant-reported data collection. We describe its user-centered development and pilot implementation within the NCI-sponsored Multi-Cancer Early Detection (MCED) Biobank Study (A212102).

**Methods:**

PEP was developed through iterative user-centered design processes that included advisory board input, usability testing with 19 patients (12 Spanish-speaking), and plain language and health-literacy refinement. PEP was introduced as an opt-in component of the MCED Biobank Study. PEP participants completed demographic and social determinants of health (SDOH) surveys. Implementation outcomes were assessed using participant and site-staff surveys.

**Results:**

Among 2221 MCED participants, 899 (40%) enrolled in PEP; 361 completed demographic items and 310 completed SDOH questions. Most participants reported a positive experience (84%), easy access (96%), and ease of survey completion (93%). PEP enrollment occurred at 154 of 630 open sites, with higher uptake in community than academic practices. Fidelity was moderate, with 38% completing baseline surveys and 32% completing SDOH items. Willingness to be re-contacted was high, with 93% agreeing to be contacted about future research and 98% open to health-related follow-up.

**Conclusions:**

A user-centered development approach produced a feasible, well-received digital engagement platform within a national oncology network. High willingness to contribute data and remain reachable suggests PEP may serve as an effective model for sustained participant engagement in cancer research.

## Introduction

Digital health technologies, which include mobile health, wearable devices, telehealth platforms, and the data infrastructure that enables their integration, are playing an increasingly important role in clinical research.^1^^,^^2^ Digital technologies can support more efficient trial processes by simplifying access to study materials, streamlining data collection, and facilitating remote monitoring.^3^ They can also help improve participant experience by enabling bidirectional communication between researchers and participants, increasing trial accessibility, and improving the quality and timeliness of information received by participants.^4-6^ The National Cancer Institute (NCI) and clinical trials networks are actively investing in digital approaches to support streamlined research protocols and broader participation across studies.^7^^,^^8^

Participant engagement in research refers to the sustained and beneficial interaction between research participants and researchers throughout a study.^9-11^ When leveraged thoughtfully, digital health technologies may help anticipate and address study challenges, foster transparency, build and repair trust, deepen understanding of contextual factors, and enhance the relevance and adoption of research findings.^12-15^ In clinical cancer research, digital technologies are among the most frequently used modes of engagement with research participants, and are used across the clinical trial lifecycle from recruitment to return of results.^16^

The Participant Engagement Portal (PEP) is a newly developed web-based tool designed to facilitate bidirectional communication between studies sponsored by Alliance for Clinical Trials in Oncology (Alliance) and study participants.^17^ PEP was initiated to help researchers learn directly from participants’ experiences to improve the conduct of future trials. It was designed to support richer engagement of participants already enrolled in a clinical trial, rather than to enhance enrollment in a trial. Alliance brings together over 10 000 cancer specialists from hospitals, medical centers, and community clinics across the United States and Canada to conduct clinical trials to advance cancer prevention, diagnosis, and treatment.

In this article, we present our user-centered design process and evaluate the implementation and early findings of PEP based on its inclusion in the national, NCI-sponsored Multi-Cancer Early Detection (MCED) Biobank Study (A212102). Information from this work can support researchers, bioinformaticians, clinical research groups, and others in designing portals dedicated to digitally engaging participants in research.

## User-centered design of PEP

PEP was designed following principles of user-centered design. In health contexts, user-centered design is a process for developing products, systems, and solutions that center on the needs and preferences of patients, providers, and other relevant stakeholders.^18^ User-centered design is often iterative and relies on the use of multiple approaches to gather insight and feedback in cycles.^19^ Approaches including expert and community engagement, usability testing, pilot testing, and survey research facilitated the design and evaluation of PEP (Table 1). Activities associated with the design of PEP were deemed exempt by Advarra, a Central IRB vendor (Protocol# DL-002). Below we describe the activities included to facilitate the user-centered design.

**Table 1.** User-centered design process of the Alliance Patient Engagement Portal (PEP).

PEP design activity	Description	Key impacts on PEP design
Engaging with an advisory board	Formation of a multidisciplinary advisory board to guide design and development of PEP	Feedback supported technical feasibility, alignment with community priorities, and strategies for clinical trial engagement
Developing content	Development of bidirectional content including trial-specific materials and participant-reported data	SDOH and demographic surveys were integrated based on expert input and used Gravity Project standards to support interoperability
Building technical infrastructure	Creation of a secure, modifiable web platform in partnership with MITRE	Existing platform capabilities enabled rapid customization and anonymous access; supported robust data collection workflows
Optimizing usability	Enhancements made for clarity, comprehension, and accessibility	Findings resulted in improved language and visual design.; bilingual options and culturally nuanced content increased accessibility and comprehension
End-user testing	Interviews with end-users to identify areas for refinement	Users feedback shaped content and design adaptations, and particularly highlighted the importance of site transparency and clarity on survey timing

SDOH Social Determinants of Health

### Engaging with an advisory board

A multidisciplinary advisory board was engaged to support the design of PEP and to contribute to the future evolution of the PEP initiative. Advisory board members included people with cancer, cancer survivors, caregivers, clinicians, clinical research personnel, principal investigators for potential partnered trials for PEP, statisticians, and experts on digital health portal development. The advisory board met every 6 months over the course of 2 years. During these meetings, the board reviewed the ongoing PEP initiative and discussed potential growth of the capabilities and functions of PEP to align with the goals for clinical trial communication, patient education, and engagement. They also advised on topics related to balancing community needs and preferences for the PEP with technical feasibility of the desired capabilities. Individual members of the advisory board were also engaged as needed to advise on specific topics.

### Selecting PEP content

Content for PEP was developed in 2 primary domains: delivery of trial-specific content and collection of participant-reported information. Trial-specific content communicated information to participants about the purpose and process of their clinical trial, updates, future clinical trials that may interest them, resources that may be helpful through their cancer treatment, and trial results. Participant-reported information allowed individuals to complete surveys and provide information on demographics and social determinants of health (SDOH). The first survey assessed demographic information and the second assessed sensitive questions about income, education, and SDOH. SDOH data were of interest given evidence of their impact on cancer patient health outcomes.^20^ SDOH questions used for PEP were selected in consultation with a social determinants of health expert and based on their content and endorsement by the Gravity Project, a public-private collaborative developing consensus-driven SDOH data standards that could allow for future data aggregation across other projects that utilize this data standard.^21^

### Establishing PEP technical infrastructure

The PEP online platform was developed in partnership with The MITRE Corporation, a federally funded research and development center.^22^ The platform was adapted from an award-winning platform developed to support public health agencies in disease tracking and symptom monitoring during the COVID-19 pandemic.^23^ It allowed for participant registration through email or text messaging; offered features including filtering and scheduling messages based on preferred times, time zones, and target populations; and was readily modifiable for PEP infrastructure and goals. It also enabled participants to access PEP surveys through a unique tokenized capability URL rather than through a login.^24^ The use of unique URLs rather than login was recommended by the advisory board and intended to reduce participant burden; username and passwords may be forgotten and create barriers that could limit uptake and sustained engagement. PEP was designed to be used on either a desktop or mobile device and included features to optimize user experience on either platform, such as automatic adjustment of screen sizes to provide a positive user experience. Within the platform, surveys were housed on Lime Survey, an open source online survey program.^25^

### Optimizing PEP usability

Proactive steps were taken to optimize the usability of PEP. First, a plain language specialist edited all written text for clarity and conciseness. Additionally, a health-literacy graphic designer advised on the PEP materials and website to improve flow and ease of comprehension including using colorful infographics and images. Content was then reviewed by patient advocates from the advisory board for feedback including timing and ease of completion of surveys. Finally, the PEP website and all related materials were made available in both English and Spanish. This process included linguistically translating the materials as well as refining them through a transcreation process to reflect cultural nuances.

**Figure 1.**
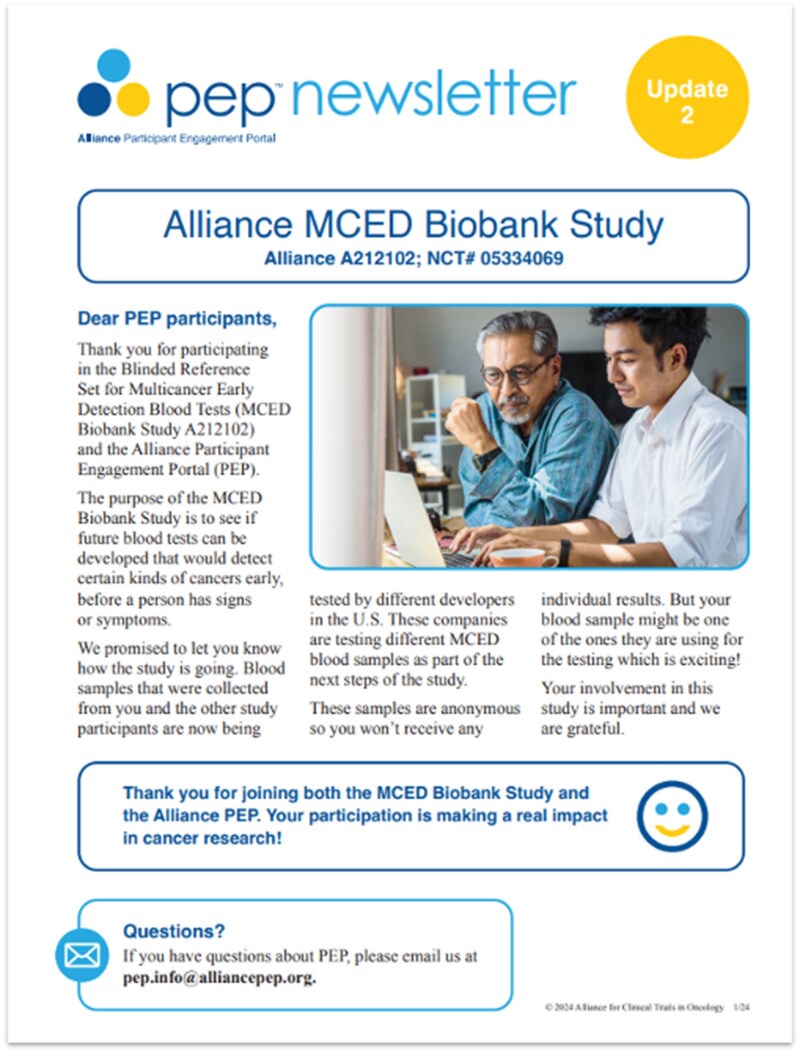
PEP newsletter.

Changes were also made to PEP in response to usability testing from end users. A series of 19 semi-structured interviews (including 12 in Spanish) were conducted with patients recruited from Alliance-affiliated medical centers by cancer treatment center staff to explore reactions to PEP materials and usefulness of the website. Individuals were eligible if they had a history of cancer, spoke English or Spanish, and were at least 18 years of age. Interview participants generally found the PEP website easy to understand, appreciated the choice of communication methods (text or email), and expressed interest in seeing survey results and knowing survey duration upfront. They also wanted more transparency about local vs national study teams and the Alliance organization to build trust, and offered some feedback to improve the Spanish-language site. Participants generally expressed that PEP was a helpful, centralized, and innovative resource, and many expressed openness to peer connections. Several participants identified gaps in information and posed clarifying questions which were used to refine the materials. A snapshot of PEP material is included in Figure 1.

## PEP pilot

A pilot was conducted to explore the implementation of PEP across Alliance sites. This piloting of PEP was approved by the National Cancer Institute Central Investigational Review Board (NCI/CIRB). PEP was rolled out as a part of Alliance protocol A212102; A Blinded Reference Set for Multicancer Detection Blood Tests (MCED Biobank Study). The MCED Biobank Study has various enrollment cohorts that include healthy individuals as well as those with cancer. It is a biospecimen study in which patients provide consent and a blood sample at a single time point.

From August 2022 to September 15, 2025, PEP was presented as an option to individuals who consented to the Alliance A212102 MCED Biobank study. To minimize administrative burden, this pilot was designed as an opt-in only. If a person chose to participate in PEP, they completed a contact card indicating interest and expressing whether they wished to receive communications by text or email. If a person did not wish to participate in PEP, no action was needed. Enrollees received a welcome message with their unique URL to invite them to PEP and complete an initial survey. Reminders were sent out at predetermined intervals to either ask them to complete the initial survey or invite them to complete subsequent surveys.

The pilot was designed as an exploratory evaluation to characterize early implementation of PEP. The pilot was evaluated in 2 ways. First, we examined the characteristics of participants who enrolled in PEP and compared them to the broader sample in the MCED Biobank Study, where PEP was embedded. Data for this analysis were collected through surveys administered via the PEP platform. Second, the implementation of PEP was assessed by evaluating its performance on implementation outcomes adapted from Proctor’s implementation outcome framework.^26^ Data for this assessment were generated from post-PEP evaluation surveys from PEP participants asking them to indicate their overall impression of PEP, whether they found PEP easy to access and complete, and whether they found information offered in PEP helpful. Data were also collected from clinical research staff surveys, which assessed workflow, perceived burden on staff members, and thoughts on MCED participants who declined PEP. As an exploratory study, no formal performance targets were pre-specified for opt-in rate, survey engagement, or compliance.

### PEP pilot participants

A total of 899 individuals signed up for PEP out of a total 2221 enrolled in the MCED Biobank Study. Of these, 361 individuals completed the first survey providing basic demographic information (Table 2). English was the primary language spoken at home for the majority (94%), and 71% were married. Survey 2 capturing SDOH was completed by 310 participants. Most participants reported completing college or a higher level of education (70%), but half (47%) had a household income below $100 000. A majority worked outside the home (71%) and had health insurance (99%). Although almost all had stable housing (97%), 14% reported challenges with their living conditions such as pests, mold, lead pipes, lack of heat, stove malfunctions, absent smoke detectors, or water leaks. Indicators of financial instability were reported by some participants: a third (33%) did not have enough money to pay bills at least occasionally, 5% worried about food running out before they could afford more, and 4% were unable to purchase needed food.

**Table 2.** Survey results from PEP pilot.

	PEP pilot
**Demographics (survey 1), No. (%)**	*n* = 361
Marital status	
Married	255 (70.6%)
Divorced/separated	46 (12.7%)
Single	29 (8.0%)
Widowed	12 (3.3%)
Prefer not to answer	8 (2.2%)
Language spoken at home: English only	339 (93.9%)
**Social determinants of health (survey 2), No. (%)**	*n*= 310
Highest level of education: college graduate or higher	216 (69.7%)
Annual household income: less than $100 000	147 (47.4%)
Works outside the home	221 (71.3%)
Has health insurance	306 (98.7%)
Living situation: steady place to live	301 (97.1%)
Has problems at living situation^a^	43 (13.9%)
Does not have enough money to pay bills at least sometimes	101 (32.6%)
Worried that food would run out before able to purchase more	15 (4.8%)
Did not have enough money to purchase needed food	12 (3.9%)
Lacks reliable transportation	9 (2.9%)
Threat of utilities being shut off	9 (2.9%)
**Indicated a willingness to…, No. (%)**	*n* = 361
Provide street address	217 (60.1%)
Provide zip code	361 (100%)
Provide information on ancestry/ethnic origin	338 (93.6%)
Be re-contacted for future research	334 (92.5%)
Be re-contacted about health	354 (98.1%)

a Includes pests, mold, lead pipes, lack of heat, stove not working, no smoke detectors, water leaks.

Participants demonstrated a strong willingness to share personal data to support research efforts. All respondents (100%) provided their zip code, and 60% provided detail down to the level of street address. Ninety-four percent of participants were comfortable disclosing ancestry or ethnic origin. Most agreed to be re-contacted for future research (93%) or for health-related follow-up (98%).

As compared to those in the MCED Biobank Study overall, PEP participants were more likely to be female (70% vs 56%, *P* < .01, Table 3) and White (84% vs 71%, *P* < .01), and less likely to be African American or Black (9% vs 17%, *P* < .01) or Hispanic (3% vs 9%, *P* < .01). MCED study and PEP pilot participants were similarly aged.

**Table 3.** Comparing characteristics of PEP pilot and overall MCED Biobank Study participants.

Characteristic	PEP pilot	MCED Biobank	*P*-value
	*n* = 361	*n* = 2221	
Female, No. (%)	252 (70)	1235 (56)	<.01
Age, years, mean (range)	57.2 (37 to 78)	58.7 (39 to 78)	<.01
Race, No. (%)			
American Indian or Alaska Native	5 (1)	35 (2)	.97
Asian	16 (4)	112 (5)	.72
Black or African American	33 (9)	378 (17)	<.01
Native Hawaiian or other Pacific Islander	2 (1)	8 (0.4)	.93
White	303 (84)	1574 (71)	<.01
Not reported	10 (3)	34 (2)	.14
Other	4 (1)	78 (7)	.02
Ethnicity: Hispanic or Latino, No. (%)	12 (3)	191 (9)	<.01

### Implementation evaluation of PEP pilot

Implementation outcomes for the PEP initiative were assessed across 6 domains: acceptability, appropriateness, adoption, feasibility, fidelity, and reach. Descriptions of these domains and their assessment are presented in Table 4.

**Table 4.** Evaluation of PEP implementation pilot.

Implementation outcome	Description	Metric	Finding
Acceptability	Perception that innovation is agreeable	Percent enrollees with a positive evaluation of PEP	84% of PEP enrollees who completed a post-PEP evaluation had an overall positive evaluation of PEP
Adoption	Proportion and representativeness of sites implementing the innovation	Proportion of study sites recruiting participants to PEP	154 out of 630 sites recruited at least 1 participant to PEP. Community sites exhibited greater adoption than academic sites
Appropriateness	Perceived fit and relevance of the innovation among enrollees	Percent enrollees indicating PEP materials were helpful	68% of PEP enrollees who completed a post-PEP indicated materials were helpful
Feasibility	Practicality of implementing the innovation	Percent enrollees indicating that PEP was easy to access and complete. Percent staff indicating that enrolling participants in PEP was easy and percent indicating that it had minimal burden on workflow	96% of PEP enrollees who completed a post-PEP evaluation thought it was easy to access, and 93% thought surveys were easy to complete 77% of staff who completed a post-PEP evaluation reported it was it was easy or somewhat easy to sign people up for PEP, and 71% replied it was no or minimal burden to sign people up
Fidelity	Degree to which innovation was implemented as it was intended	Percent of PEP enrollees completing each of the 3 surveys	38% of PEP enrollees completed the first survey on demographics, and 32% completed the second survey on SDOH
Reach	Proportion and representativeness of eligible individuals participating in the innovation	Percent of eligible individuals enrolled and comparison of enrollee sociodemographic to broader MCED Biobank Study	41% of eligible MCED Biobank Study participants enrolled in PEP; Variability in reach varied across sites, with trend for community sites to have higher reach than academic sites

Eighty-four percent of PEP enrollees indicated that participating in PEP was a positive experience. Participants also found PEP to be appropriate, with 68% indicating that the content was relevant and helpful. Positive comments from participants included that individuals were happy to help advance cancer research and enjoyed receiving messages and reading about the trial.

As of November 2024, the MCED Biobank Study protocol was open at 630 sites, 154 of which had enrolled at least 1 patient into PEP. Site enrollments ranged from 1 to 33 people per individual site. PEP recruitment varied across sites. For instance, 1 community site enrolled 34 patients into the MCED Biobank Study, of which 33 also enrolled in PEP (97%), whereas a larger academic site enrolled 57 patients in the biobanking study, only 7 of whom also enrolled in PEP (12%). Overall, the community sites tended to enroll a higher percentage of overall trial patients into PEP vs the larger academic sites.

PEP also showed feasibility as a method for engagement. Among enrollees who completed the PEP evaluation survey, 96% reported that the program was easy to access, and 93% found the surveys easy to complete. Staff perspectives were similarly favorable, with 77% indicating that enrolling participants was easy or somewhat easy, and 71% reporting that the process imposed no or minimal burden on their workflow.

Fidelity to the intended protocol was moderate. Among those enrolled, 38% completed the baseline demographics survey and 32% completed the subsequent survey on social determinants of health. Reach was also moderate, with only 41% of eligible participants from the broader MCED Biobank Study enrolling in the program. Additionally, participants in PEP were more likely to be female and White as compared to those participants in the broader MCED Biobank Study. Only 8 individuals registered using the Spanish PEP platform. The reasons individuals declined PEP, as reported by site staff, were largely that they are overwhelmed with a cancer diagnosis (39%) or did not wish to have more information about the MCED Biobank Study (48%).

## Discussion

This study demonstrates a successful user-centered design process and pilot of PEP within the context of cancer clinical research. The process prioritized stakeholder feedback and iterative refinements to create a digital health resource that was accessible, informative, and engaging for patients participating in trials.

The user-centered design of PEP was informed by expert consultation, advisory board input, interviews with diverse patients, and usability best practices. Acceptability among participants was high, with positive feedback on the bidirectional communication model, multilingual accessibility, and ease of comprehension. Despite general satisfaction with PEP expressed during both development and implementation, feedback also identified opportunities for improvement, including greater transparency and refinement of Spanish-language materials. Improvements in PEP were made iteratively and in response to feedback and aimed to meet end-user preferences while also working within the system's functionality. Continuous feedback loops informed enhancements such as a redesign of the PEP resource page, with follow-up surveys validating the helpfulness of new additions.

High rates of acceptability for future health communications (97%) and research follow-up (93%) hold promise for scaling PEPs use across additional Alliance trials. This future contact permission is a critical success of the PEP surveys as the potential is there for re-engagement of this population. Through PEP presentations at clinician meetings, social media posts, and research site PEP webinars, there is increased awareness of PEP across Alliance. As PEP has been refined in content and workflow, more investigators are inquiring about partnering their clinical study with PEP. Among those enrolled in the MCED Biobank Study, 40% opted into PEP, and of those, 40% completed the first survey and 86% went on to complete the second. This suggests that once participants engaged meaningfully with the platform, they were likely to continue using it. Situating these rates within the existing literature is difficult, as reported engagement metrics vary considerably across studies depending on population, enrollment design, and context.^27^^,^^28^ These pilot findings provide a useful early reference point for what opt-in digital engagement looks like within a large national oncology network, and can help inform expectations and design decisions for future iterations of PEP and similar platforms.

PEP adoption varied meaningfully across sites, with community sites enrolling a higher proportion of eligible participants into PEP than academic institutions. Prior work on digital health tools and clinical trial engagement suggests that site level variation is common and often reflects differences across a myriad of factors including patient populations, research infrastructure, and study staff processes.^29-31^ Differences observed in the present study likely reflect a combination of factors not directly measured in this evaluation but potentially including differences in patient populations and outreach and enrollment practices by study staff. These differences suggest a need for site-specific implementation strategies of PEP that can be tailored both based on participant and site factors. In PEP surveying, 75% of site study staff reported not having viewed the PEP website themselves, underscoring the need for more robust site staff engagement. Future iterations could include strategies to actively engage clinical site staff such as through structured briefings, integration into onboarding protocols, and proactive sharing of PEP materials. An electronic registration workflow is currently under investigation as an alternative to the contact form to reduce administrative burden, and site educational training videos are planned through the Clinical Trials Support Unit eLearning portal.

Several considerations are relevant when interpreting the generalizability of our pilot PEP findings. PEP used an opt-in approach for participation among a cohort of healthy volunteers. As a result, the current sample may underrepresent engagement levels in cancer-patient populations, possibly yielding conservative estimates of uptake. The opt-in approach also introduces potential selection bias. PEP enrollees skewed more female and White compared to the overall MCED Biobank Study population in which PEP was piloted. This aligns with trends in digital health engagement.^32^^,^^33^ Age distributions were similar across the MCED Biobank Study, suggesting that PEP effectively reached target age groups. Additionally, as a digital health tool requiring access to internet devices, the study may have preferentially engaged individuals people who have greater interest in or capacity to use digital tools for health purposes. Taken together, these considerations indicate that early engagement results reflect the experiences of a subset of enrolled participants rather than the broader population.

Future work should prioritize strategies to improve reach and representativeness, including exploring opt-out enrollment approaches, and increasing engagement with site personnel. PEP was designed to support engagement among individuals already enrolled in the parent trial and was not intended to influence trial enrollment. Our findings speak to the potential of the platform to support participants who have already enrolled in clinical trials, rather than those who may be more broadly eligible. Achieving more representative enrollment is a critical upstream priority for cancer clinical trials generally.^34-37^ Building on the strong willingness to contribute data and remain reachable demonstrated by PEP participants, future efforts should also focus on formalizing recontact pathways within trial networks and enhancing tracking of opt-outs and passive disengagement. PEP demonstrates a model for integrating digital tools in cancer research through the combination of technological innovation with user-centered design.

## Data Availability

Data may be available from Alliance upon reasonable request.
